# MRI-assessed tumor-free distance to serosa predicts deep myometrial invasion and poor outcome in endometrial cancer

**DOI:** 10.1186/s13244-021-01133-z

**Published:** 2022-01-08

**Authors:** Julie Andrea Dybvik, Kristine E. Fasmer, Sigmund Ytre-Hauge, Jenny Hild Aase Husby, Øyvind O. Salvesen, Ingunn Marie Stefansson, Camilla Krakstad, Jone Trovik, Ingfrid S. Haldorsen

**Affiliations:** 1 Mohn Medical Imaging and Visualization Centre (MMIV), Department of Radiology, Haukeland University Hospital, Post Office Box 1400, 5021 Bergen, Norway; 2 Section for Radiology, Department of Clinical Medicine, University of Bergen, Jonas Lies vei 87, 5021 Bergen, Norway; 3 Unit for Applied Clinical Research, Department of Public Health and Nursing, Norwegian University of Science and Technology, Post Office Box 8905, 7491 Trondheim, Norway; 4 Department of Pathology, Haukeland University Hospital, Post Office Box 1400, 5021 Bergen, Norway; 5 Centre for Cancer Biomarkers, Department of Clinical Medicine, University of Bergen, Jonas Lies vei 87, 5021 Bergen, Norway; 6 Department of Obstetrics and Gynaecology, Haukeland University Hospital, Post Office Box 1400, 5021 Bergen, Norway; 7 Centre for Cancer Biomarkers, Department of Clinical Science, University of Bergen, Post Office Box 7804, 5020 Bergen, Norway

**Keywords:** Endometrial neoplasm, Magnetic resonance imaging, Radiologists, Biomarkers, Progression-free survival

## Abstract

**Objectives:**

To explore the diagnostic accuracy of preoperative magnetic resonance imaging (MRI)-derived tumor measurements for the prediction of histopathological deep (≥ 50%) myometrial invasion (pDMI) and prognostication in endometrial cancer (EC).

**Methods:**

Preoperative pelvic MRI of 357 included patients with histologically confirmed EC were read independently by three radiologists blinded to clinical information. The radiologists recorded imaging findings (T1 post-contrast sequence) suggesting deep (≥ 50%) myometrial invasion (iDMI) and measured anteroposterior tumor diameter (APD), depth of myometrial tumor invasion (DOI) and tumor-free distance to serosa (iTFD). Receiver operating characteristic (ROC) curves for the prediction of pDMI were plotted for the different MRI measurements. The predictive and prognostic value of the MRI measurements was analyzed using logistic regression and Cox proportional hazard model.

**Results:**

iTFD yielded highest area under the ROC curve (AUC) for the prediction of pDMI with an AUC of 0.82, whereas DOI, APD and iDMI yielded AUCs of 0.74, 0.81 and 0.74, respectively. Multivariate analysis for predicting pDMI yielded highest predictive value of iTFD <  6 mm with OR of 5.8 (*p* < 0.001) and lower figures for DOI ≥ 5 mm (OR = 2.8, *p* = 0.01), APD ≥ 17 mm (OR = 2.8, *p* < 0.001) and iDMI (OR = 1.1, *p* = 0.82). Patients with iTFD < 6 mm also had significantly reduced progression-free survival with hazard ratio of 2.4 (*p* < 0.001).

**Conclusion:**

For predicting pDMI, iTFD yielded best diagnostic performance and iTFD < 6 mm outperformed other cutoff-based imaging markers and conventional subjective assessment of deep myometrial invasion (iDMI) for diagnosing pDMI. Thus, iTFD at MRI represents a promising preoperative imaging biomarker that may aid in predicting pDMI and high-risk disease in EC.

**Supplementary Information:**

The online version contains supplementary material available at 10.1186/s13244-021-01133-z.

## Key points

- Tumorfree distance to serosa (iTFD) at preoperative MRI represents a promising preoperative imaging biomarker.
- iTFD yielded highest AUC for the prediction of histopathological deep myometrial invasion.
- Interobserver agreement for assessing iTFD < 6 mm at preoperative MRI was moderate.

## Introduction

Endometrial cancer (EC) is the sixth most common neoplasm in women worldwide, and the incidence has been increasing over the past decades [1, 2]. EC is surgicopathologically staged according to the International Federation of Gynecology and Obstetrics (FIGO) staging system. Surgical treatment is normally individualized based on putative risk profile. Primary treatment consists of simple total hysterectomy and bilateral salpingo-oophorectomy in patients assumed to have low FIGO stage and low-risk histological subtype. In patients with putative advanced FIGO stage, high-risk histological subtype and/or hormone receptor loss, surgical treatment may include radical hysterectomy (if suspected cervical stroma invasion) and/or pelvic and/or paraaortic lymphadenectomy or lymph node dissection [3, 4]. However, lymphadenectomy can also cause unfavorable side effects such as lower-extremity lymphedema and lymphocele development [5, 6], and there is therefore an unmet need for preoperative methods that identify which patients that are likely to benefit from these procedures.

Predicting the presence of pathologic deep (≥ 50%) myometrial invasion (pDMI) preoperatively is important as this surgicopathological staging feature is known to be associated with increased risk of lymph node metastasis and poor outcome in EC [7]. Thus, an accurate and reproducible imaging method for identifying pDMI is needed, if this imaging approach is to safely guide risk-stratified surgical treatment algorithms that tailor lymphadenectomy to putative high-risk patients only.

Preoperative pelvic MRI is widely used and considered the best preoperative imaging method for local staging in EC [8]. However, variable accuracy and moderate interobserver agreement between radiologists for the MRI-based staging parameter deep myometrial invasion (iDMI) have been reported [8–10]. Thus, more accurate and robust imaging markers with which to predict the presence of pDMI are highly needed [9, 11].

The primary objectives of this study were to assess the diagnostic accuracy and interobserver agreement for diagnosing deep myometrial invasion based on conventional MRI reading (iDMI) and when based on MRI tumor measurements for predicting pathologic deep myometrial invasion (pDMI) and aggressive disease in endometrial cancer patients.

## Material and methods

### Patients and study setting

This retrospective study was conducted under institutional review board approval, with written informed consent from all patients, and approved by the Regional Committee for Medical Research Ethics (REK 2015/2333, 2019/1020 and 2019/1907). From April 2009 to December 2016, preoperative pelvic MRI was performed in 357 prospectively included patients with histologically confirmed endometrial cancer. The diagnosis was established through preoperative endometrial biopsy/curettage and histologically verified in hysterectomy specimen, and the patients formed a consecutive series. All patients were diagnosed and treated at the same university hospital serving a population of approximately one million inhabitants.

Clinical data (e.g., age, menopausal status, height, body weight) were registered, and follow-up data regarding time of progression and survival were collected from patient records or correspondence with the responsible gynecologist or primary physician. Histopathologic features (histological type, grade, myometrial invasion (< / ≥ 50%), tumor-free distance to serosa in hysterectomy specimen (pTFD), cervical stroma invasion and lymph node affection) were obtained from routine pathology reports, according to published guidelines [12]. pTFD was reported in the routine pathology report in 230 patients; macroscopic pTFD (macro) in 210 patients and/or microscopic pTFD (micro) in 85 patients. When both pTFD (macro) and pTFD (micro) were reported (*n* = 65), pTFD (micro) was recorded as pTFD. The median follow-up time was 77 months (mean 76, range 0–135). Progression was defined as local recurrence or progression in the pelvis, abdomen or at distant sites.

### Imaging protocol

Pelvic MRI was performed on a 1.5 T Siemens Avanto running Syngo MR B17 (Erlangen, Germany), using a six-channel body coil, in 286/357 (80%) of the patients. 3 T Siemens Skyra running Syngo MR E11 (Erlangen, Germany) with an 18-channel body-phased array and a spine coil was used in 71/357 (20%) of the patients (Additional file 1: Supplementary table 1). Prior to imaging, 20 mg butylscopolamine bromide (Buscopan, Boehringer Ingelheim, Germany) was administered intramuscularly/intravenously in order to reduce bowel peristalsis. The MRI protocols are in line with the guidelines from the European Society of Urogenital Imaging (ESUR) [13, 14]. Gadolinium-enhanced axial oblique (relative to the long axis of the uterine body) T1-weighted images were acquired 2 min after contrast injection and axial oblique pelvic diffusion-weighted imaging (DWI) was acquired with *b*-values of 0 and 1000 s/mm^2^, and apparent diffusion coefficient (ADC) maps were generated (Additional file 1: Supplementary table 1). Median (range) interval between MRI examination and surgical staging was 9 (0–98) days.

**Table 1:** Patient demographics and tumor characteristics in the endometrial cancer study cohort (*n* = 357)

	Entire cohort	Dichotomized with iTFD-cutoff < / ≥ 6 mm		
		iTFD ≥ 6 mm	iTFD < 6 mm	*p* value^α^
Age, median (range), years	67 (30–93)	64 (32–93)	70 (30–89)	< 0.001
BMI, median (range), kg/m^2^	27 (16–53)	28 (16–53)	27 (16–50)	0.03
Postmenopausal, *n* (%)	327 (92)	171 (87)	156 (98)	< 0.001
FIGO stage, *n* (%)				**0.003**
1 & 2	307 (86)	179 (91)	128 (80)	
3 & 4	50 (14)	18 (9)	32 (20)	
Myometrial invasion, *n* (%)*				** < 0.001**
< 50%	211 (60)	162 (83)	49 (32)	
≥ 50%	139 (40)	33 (17)	106 (68)	
Cervical stroma invasion, *n* (%)*				**0.001**
No	299 (85)	177 (91)	122 (79)	
Yes	51 (15)	18 (9)	33 (21)	
Lymph node metastasis, *n* (%)**				0.16
No	213 (87)	112 (90)	101 (83)	
Yes	33 (13)	13 (10)	20 (17)	
Histological type, *n* (%)				0.79
Endometrioid	290 (81)	161 (82)	129 (81)	
Non-endometrioid		67 (19)	36 (18)	31 (19)
Histological grade in endometrioid tumors, *n* (%)***				** < 0.001**
Grade 1	160 (56)	102 (65)	58 (45)	
Grade 2	77 (27)	42 (27)	35 (27)	
Grade 3	50 (17)	14 (9)	36 (28)	

All significant *p* values are given in boldface

BMI, body mass index; FIGO, International Federation of Gynecology and Obstetrics; iTFD, tumor-free distance to serosa based on imaging findings

*Missing information in seven patients who did not undergo hysterectomy

**Missing information in 111 patients who did not undergo lymphadenectomy

***Missing information on tumor grade in three patients

^α^*p* values (asymptotic) refer to Pearson Chi-squared test for categorical variables and Mann–Whitney *U*-test for continuous variables

### Data analysis

**Fig. 1:**
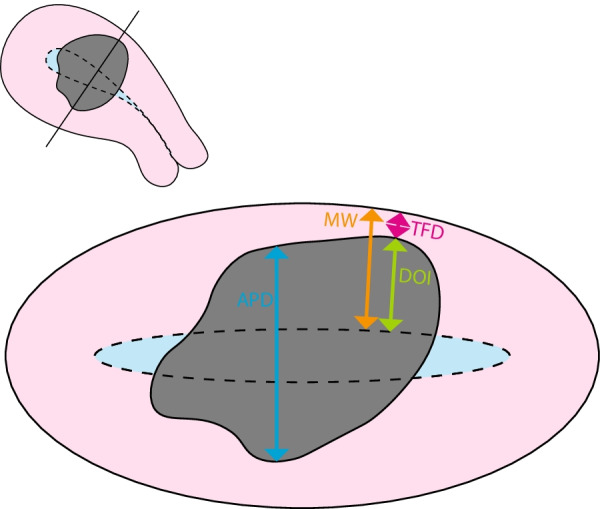
Schematic overview of the different measurements marked on an axial oblique slice of the uterus. The myometrial wall is colored pink, tumor is gray and the endometrium/uterine cavity is light blue and the boundary between the myometrium and endometrium is delineated with a dotted black line. APD (blue arrow): maximum anteroposterior tumor diameter. MW (orange arrow): presumed thickness of the myometrial wall. DOI (green arrow): absolute depth of myometrial tumor invasion in the region exhibiting proportionally deepest invasion. TFD (pink arrow): tumor-free distance to serosa in the area exhibiting deepest invasion or shortest distance to serosa. The conventional dichotomous imaging parameter, iDMI, myometrial invasion of ≥ 50% of the myometrial wall is determined by DOI relative to MW

**Fig. 2:**
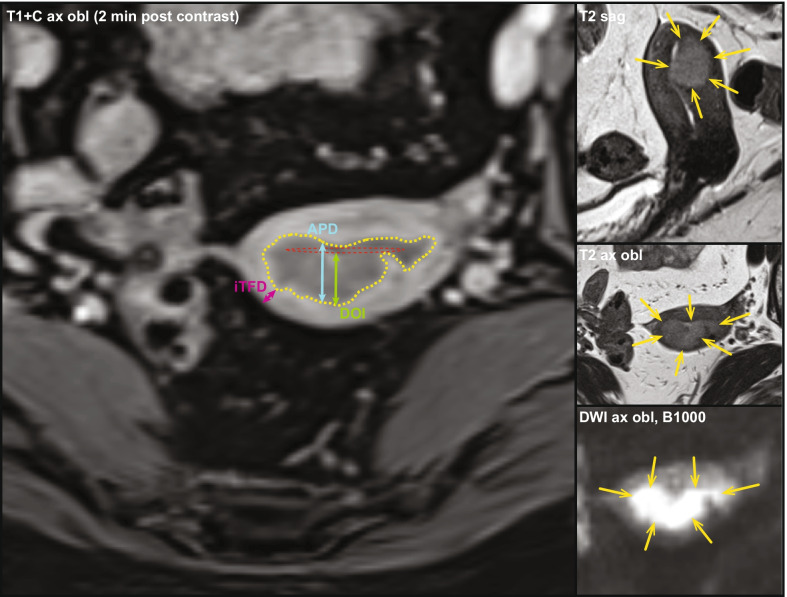
Pelvic 3 T MRI of a 68-year-old patient with endometrial cancer, FIGO stage 1B (≥ 50% myometrial invasion). Tumor was MRI-staged to iDMI (≥ 50%), maximum anteroposterior tumor diameter (APD) 17 mm, maximum depth of myometrial invasion (DOI) 7 mm and tumor-free distance to serosa (iTFD) 5 mm. The different MRI measurements are marked on an axial oblique contrast-enhanced T1-weighted image, 2 min after contrast injection. The measurements are supported by sagittal and axial oblique T2-weighted images (T2 sag and T2 ax obl) and axial oblique diffusion-weighted B-1000 image depicting restricted diffusion in the tumor. Tumor is marked with yellow arrows and yellow dotted line. Red oval line = presumed lining of the uterine cavity, green arrows = DOI, pink arrows = iTFD and blue arrows = APD

The MRI examinations were de-identified and read independently by three different radiologists who were blinded to clinical information. Each of the examinations were read by three radiologists, and in total, five radiologists with 2–10 years of experience with pelvic MRI participated in the reading of the examinations. All readers reported imaging findings in a standardized registration form, and consensus variables were generated: For categorical variables, the category recorded by the majority of the three raters was used, and for continuous variables, median values were used. Contrast-enhanced axial oblique T1-weighted images (2 min after contrast injection) were used to measure maximum axial anteroposterior tumor diameter (APD), maximum depth of myometrial invasion (DOI) and tumor-free distance to serosa (iTFD) and to assess the presence of deep (≥ 50%) myometrial invasion based on imaging findings (iDMI) (Fig. 1 and Additional file 1: Supplementary table 2). The other MRI sequences (DWI- and T2-weighted sequences) were also available aiding in the discrimination between tumor tissue and healthy normal tissue (Fig. 2). iTFD and DOI were measured in the areas exhibiting shortest distance to serosa and deepest myometrial tumor infiltration, respectively. In cases with variable myometrial thickness due to, e.g., leiomyomas in the region of the tumor, the myometrial thickness in the area abutting the leiomyoma, assumed to be more representative of the “true” myometrial thickness, was measured to decide on the presence of iDMI. The reference standard was surgicopathological FIGO stage (2009) [15], and pathologic deep (≥ 50%) myometrial invasion (pDMI) in the hysterectomy specimen was evaluated by the pathologists using standard procedures.

**Table 2:** Sensitivity, specificity, accuracy, LR + , LR- and OR for the prediction of pDMI by the preoperative MRI markers iTFD < 6 mm, DOI ≥ 5 mm, APD ≥ 17 mm and iDMI

	iTFD < 6 mm*	DOI ≥ 5 mm*	APD ≥ 17 mm*	iDMI^+^	*p* value ^α^
Sensitivity, % (no. of patients) [CI]^^^	76% (106/139) [68%-83%]	86% (119/139) [79%-91%]	78% (109/139) [71%-85%]	76% (106/139) [68%-83%]	*p*** = 0.049**^**θ**^
Specificity % (no. of patients) [CI]^^^	77% (162/211) [70%-82%]	49% (103/211) [42%-56%]	69% (146/211) [62%-75%]	73% (153/211) [66%-78%]	*p*** < 0.001**^**β**^
Accuracy [CI]^^^	77% (268/350) [72%-81%]	63% (222/350) [58%-68%]	73% (255/350) [68%-77%]	74% (259/350) [69%-79%]	*p*** < 0.001**^**λ**^
LR +	3.28	1.67	2.54	2.77	
LR-	0.31	0.29	0.31	0.33	
OR (CI)[*p* value^†^]	10.6 (6.4, 17.6) [**< 0.001]**	5.7 (3.3, 9.8) [**< 0.001]**	8.2 (5.0, 13.4) [**< 0.001]**	8.5 (5.2, 13.9) [**< 0.001]**	
OR (CI) [*p* value^†^], adjusting for histological risk^‡^	10.4 (6.3, 17.4) [**< 0.001]**	6.1 (3.5, 10.7) **[< 0.001]**	7.8 (4.7, 13.0) **[< 0.001]**	8.5 (5.2, 14.0) **[< 0.001]**	
OR (CI) [*p* value^†^] adjusting for histological risk^‡^ and all listed MRI variables	5.8 (2.9, 11.6) **[< 0.001]**	2.8 (1.3, 5.7) **[0.01]**	2.8 (1.5, 5.2) **[< 0.001]**	1.1 (0.5, 2.4) [0.82]	

APD, anteroposterior tumor diameter; CI, 95% Confidence interval; DOI, depth of invasion; iDMI, deep myometrial invasion (DMI) based on imaging findings; LR + , likelihood ratio for positive results: LR +  = sensitivity/(1-specificity); LR-, likelihood ratio for negative results: LR- = (1-sensitivity)/specificity; OR, odds ratio; pDMI, DMI based on pathology findings; iTFD, tumor-free distance to serosa based on imaging findings

*Optimal cutoff values for iTFD, DOI and APD based on the receiver operating characteristics analysis (Youden index) for the prediction of pDMI in hysterectomy specimen

^+^Deep (≥ 50%) myometrial invasion based on standard imaging reading

^α^Cochrans Q-test

^Clopper–Pearson confidence interval for proportion

^θ^DOI yields significantly higher sensitivity than iDMI (*p* = 0.02, McNemar test)

^β^iTFD yields significantly higher specificity than DOI (*p* < 0.001) and APD (*p* = 0.045, McNemar test)

^**λ**^iTFD yields significantly higher accuracy than DOI (*p* < 0.001, McNemar test)

^†^Binary logistic regression analysis

^‡^High-risk histological subtype (endometrioid grade 3 or non-endometrioid) versus low-risk histological subtype (endometrioid grade 1–2) based on preoperative endometrial biopsy/curettage

All significant *p* values are given in boldface

### Statistical analysis

The Shapiro–Wilk test of normality was used to assess normal distribution of the continuous data. Receiver operating characteristic (ROC) curves for the prediction of pDMI were plotted for the different MRI measurements, and optimal cutoff values were determined for which the best separation of Youden index was achieved. The prognostic value of the tumor measurements was analyzed using univariable Cox proportional hazard model and in multivariable models adjusting for age and high-risk histology. The concordance index was used to assess the predictive ability of all models. Differences in sensitivity, specificity and accuracy between the different tumor measurement cutoffs and iDMI for the prediction of pDMI were explored using Cochrans exact Q-test and, if significant, by pairwise analysis with McNemars test. The tests were performed using SPSS version 26.

Interobserver agreement was assessed using overall kappa (κ) and intraclass correlation coefficient (ICC) with 95% confidence interval (CI) using mixed linear model for calculation of ICC for the continuous variables, and overall kappa was estimated using the mean pairwise kappa for the dichotomous variables for all possible pair of readers; the CIs were calculated using bootstrapping. Differences between examinations performed at 1.5 T versus 3 T in sensitivity, specificity and accuracy for the different tumor measurement cutoffs and iDMI for the prediction of pDMI were explored using Fishers exact test. These analyses were performed using R (R, version 4.0.3).

Comparison between tumor-free distance reported in routine pathology report (pTFD) and based on imaging findings (iTFD) was performed using Bland–Altman plot in R (R, version 4.0.3).

All reported *p* values were two-sided, and *p* values less than 0.05 were considered statistically significant.

## Results

### Patients and treatment

Median patient age in this study cohort (*n* = 357) was 67 years. Surgicopathological FIGO stage was stage 1A (< 50% myometrial invasion) in 55% (196/357), stage 1B (≥ 50% myometrial invasion) in 24% (85/357), stage 2 (cervical stroma invasion) in 8% (28/357), stage 3 (local or regional tumor spread) in 11% (39/357) and stage 4 (growth into the rectum/bladder or distant spread) in 3% (9/357) (Table 1). Altogether, 98% (350/357) underwent primary surgical resection with hysterectomy and bilateral salpingo-oophorectomy. The remaining seven patients underwent fertility-sparing treatment (*n* = 2; both presumed FIGO stage 1) and tumor reductive surgery (*n* = 1; presumed stage 4) or were deemed medically ineligible for surgery (*n* = 4; all presumed stage 4). Lymphadenectomy or lymph node sampling was performed in 69% (246/357) of the patients. Adjuvant therapy was given to 34% (121/357), consisting of chemotherapy in 30% (107/357), pelvic radiation therapy in 3% (11/357) and hormonal therapy in 1% (3/357).

### Imaging markers for the prediction of pDMI

**Fig. 3:**
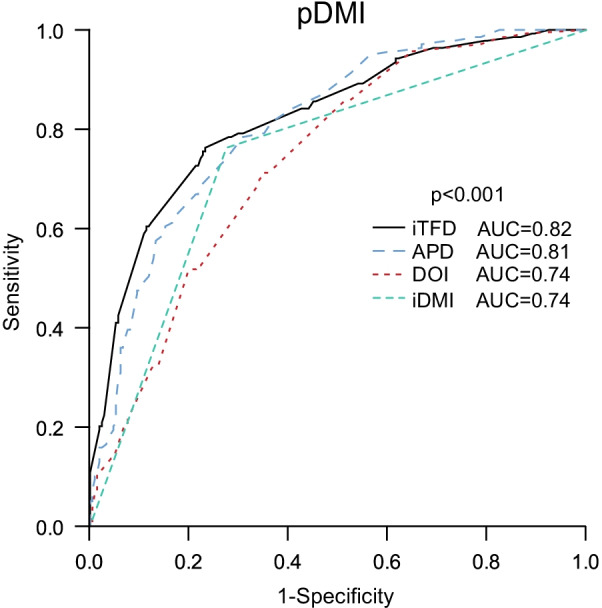
Receiver operating characteristic (ROC) curves for the different MRI tumor measurements for the prediction of pDMI (surgicopathologically deep myometrial invasion). APD (anteroposterior diameter), DOI (depth of myometrial invasion), iDMI (presence of deep (≥ 50%) myometrial invasion based on standard imaging reading) and iTFD (tumor-free distance to serosa based on imaging findings). *p* value refers to the test of equal AUC values across the different tumor measurements

For preoperative prediction of pDMI based on MRI-derived imaging markers, iTFD yielded the highest area under the ROC curve (AUC) with an AUC of 0.82, whereas DOI, APD and iDMI yielded AUCs of 0.74, 0.81 and 0.74, respectively (Fig. 3). The optimal cutoff values for the prediction of pDMI by the different preoperative MRI measurements were: iTFD < 6 mm, DOI ≥ 5 mm and APD ≥ 17 mm yielding corresponding odds ratios (ORs) of 10.6, 5.7 and 8.2 respectively (*p* < 0.001 for all) for the prediction of pDMI (Table 2). The variable iDMI (based on conventional reading) yielded an OR of 8.5 (*p* < 0.001) for the prediction of pDMI. In multivariate analysis including all cutoff-based imaging variables, iDMI and histological risk status (high-risk [endometrioid grade 3 or non-endometrioid histology] and low-risk [endometrioid grade 1–2] based on preoperative biopsy/curettage), iTFD < 6 mm yielded the highest predictive value with an OR of 5.8 (*p* < 0.001) (Table 2). APD ≥ 17 mm and DOI ≥ 5 mm also independently predicted pDMI (OR = 2.8; *p* < 0.001 and OR = 2.8; *p* = 0.01, respectively), while iDMI did not (OR = 1.1; *p* = 0.82) (Table 2). Patients with iTFD < 6 mm were typically older and more often diagnosed with pDMI, cervical stroma invasion and grade 3 endometrioid tumors than patients with iTFD ≥ 6 mm, whereas no difference in prevalence of lymph node metastases was observed for patients with iTFD < / ≥ 6 mm (Table 1).

The MRI-derived imaging markers yielded significantly different sensitivities (*p* = 0.049), specificities (*p* < 0.001) and accuracies (*p* < 0.001) for diagnosing pDMI (Table 2). DOI ≥ 5 mm yielded highest sensitivity (86%), whereas iTFD < 6 mm yielded highest specificity (77%) and accuracy (77%). DOI ≥ 5 mm yielded significantly higher sensitivity than iDMI (*p* = 0.02). iTFD < 6 mm yielded significantly higher specificity than both APD ≥ 17 mm and DOI ≥ 5 mm (*p* = 0.045 and *p* < 0.001, respectively), and DOI ≥ 5 mm yielded significantly lower specificity (*p* < 0.001) and accuracy (*p* ≤ 0.001) compared to all of the other MRI measurements for predicting pDMI (Additional file 1: Supplementary table 3).

**Table 3:** Univariable and multivariable Cox regression and concordance analyses for the prediction of progression-free survival by MRI variables in 357 endometrial cancer patients

Imaging variables	Univariable model		Multivariable model ^α^	
	HR (95%CI) [*p* value]*	Concordance	HR (95% CI) [*p* value]* ^α^	Concordance
iTFD (< 6 mm)	**2.36 (1.48–3.76) *p* < 0.001**	0.60	**1.96 (1.20–3.18) *p* = 0.007**	0.72
DOI (≥ 5 mm)	**2.19 (1.26–3.80) *p* = 0.005**	0.58	**2.10 (1.21–3.66) *p* = 0.009**	0.72
APD (≥ 17 mm)	**2.77 (1.69–4.55) *p* < 0.001**	0.63	**2.17 (1.31–3.59) *p* = 0.003**	0.72
iDMI	**2.95 (1.81–4.82) *p* < 0.001**	0.63	**2.74 (1.66–4.51) *p* < 0.001**	0.73
Patient age (yrs)	**1.04 (1.01–1.06) *p* = 0.003**	0.61		
Preoperative high-risk histology ^β^	**4.14 (2.62–6.53) *p* < 0.001**	0.66		

APD, anteroposterior tumor diameter; CI, confidence interval; DOI, depth of invasion; HR, hazard ratio; iDMI, deep (≥ 50%) myometrial invasion based on standard imaging reading; iTFD, tumor-free distance to serosa based on imaging findings

*Cox proportional hazard model, *p* value refers to log-rank test

^α^Multivariable analyses for each imaging variable after adjusting for patient age and preoperative high-risk histology

^β^High-risk histology (endometrioid grade 3/non-endometrioid histology) based on preoperative curettage/biopsy

All significant *p* values are given in boldface

When comparing the diagnostic accuracies for predicting pDMI based on 1.5 T versus 3 T MRI, there were no significant difference in specificities and accuracies for the different MRI imaging markers (Additional file 1: Supplementary table 4). However, 3 T MRI yielded higher sensitivities of DOI ≥ 5 mm and iDMI for predicting pDMI than 1.5 T MRI (100% vs. 81% [*p* = 0.004] and 91% vs. 72% [*p* = 0.02], respectively; Additional file 1: Supplementary table 4).

**Table 4:** Interobserver agreement between three readers for the recorded preoperative continuous and dichotomous imaging variables in 357 endometrial cancer patients

Continuous variables	ICC (95% CI**)
iTFD (mm)	0.73 (0.67–0.77)
DOI (mm)	0.37 (0.30–0.43)
APD (mm)	0.87 (0.80–0.91)

Dichotomous variables	Overall kappa (95% CI)
iTFD < 6 mm	0.59 (0.52–0.65)
DOI ≥ 5 mm	0.29 (0.22–0.36)
APD ≥ 17 mm	0.80 (0.75–0.85
iDMI	0.41 (0.34–0.47)

iTFD, tumor-free distance to serosa based on imaging findings; DOI, depth of invasion; APD, anteroposterior tumor diameter; iDMI, deep (≥ 50%) myometrial invasion based on standard imaging reading; CI, confidence interval; ICC, intraclass correlation coefficient, estimated using mixed linear model

**Estimated using bootstrapping

Overall kappa was estimated using the mean pairwise kappa of all possible pairs of readers

### Comparison of pTFD and iTFD

**Fig. 4:**
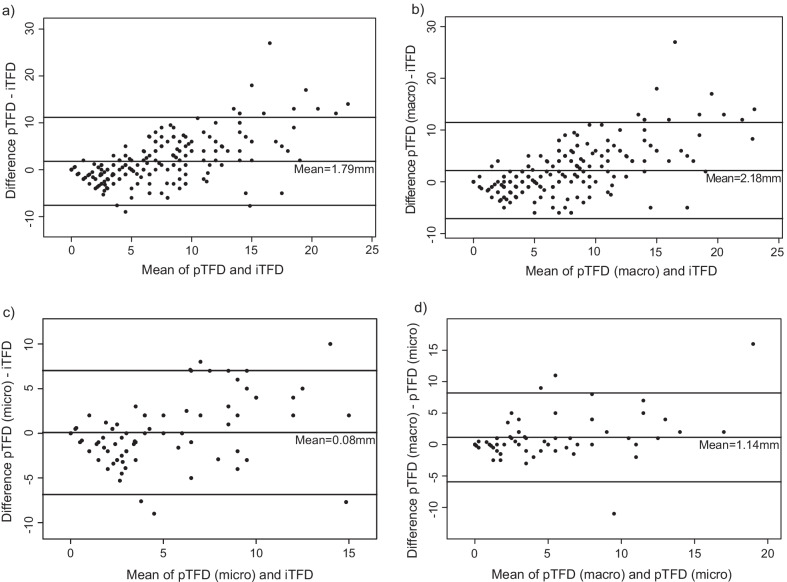
Bland–Altman plots depicting the differences between TFD measured in hysterectomy specimen (pTFD) versus by MRI (iTFD) showing best agreement between pTFD and iTFD for the low TFD values (**a**-**c**). Mean pTFD (*n* = 230) was 1.79 mm larger than mean iTFD (**a**); when based on macroscopic assessment mean pTFD (macro) (*n* = 210) was 2.18 mm larger than iTFD (**b**) and when based on microscopic assessment mean pTFD (micro) (*n* = 85) was 0.08 mm larger than mean iTFD (**c**). In patients having recordings on both pTFD (macro) and pTFD (micro) (*n* = 65), mean pTFD (macro) was 1.14 mm larger than mean pTFD (micro) (**d**). pTFD = tumor-free distance reported in routine pathology report; iTFD = MRI-assessed tumor-free distance to serosa; pTFD (macro) = tumor-free distance to serosa based on macroscopic assessment; pTFD (micro) = tumor-free distance to serosa based on microscopic assessment; SD = standard deviation

The agreement between pTFD (*n* = 230) and iTFD was good with an ICC of 0.75. Corresponding ICCs for pTFD (macro) (*n* = 210)/pTFD (micro) (*n* = 85) and iTFD were 0.76/0.78. Best agreement between pTFD and iTFD was observed for the low TFD values (Fig. 4a–c), and the agreement between pTFD (micro) and iTFD was the best with a mean difference between pTFD (micro) and iTFD of only 0.08 mm (Fig. 4c).

### Imaging markers for the prediction of survival

**Fig. 5:**
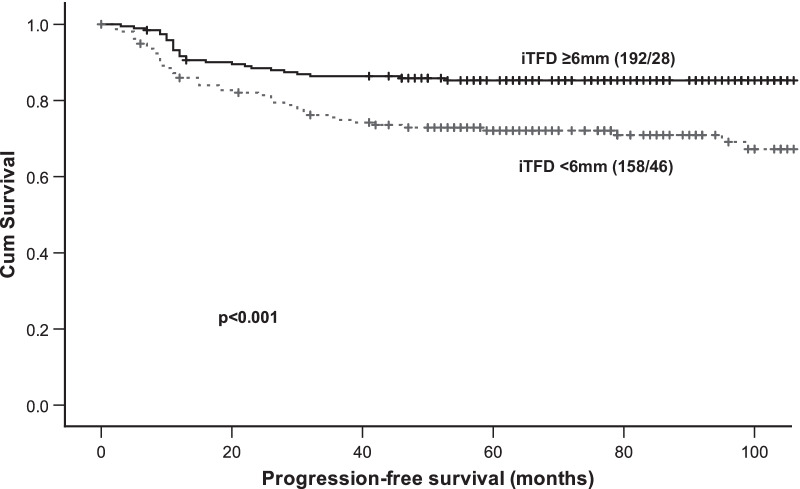
Kaplan–Meier plot depicting progression-free survival according to MRI measured iTFD (tumor-free distance to serosa based on imaging findings) ≥ 6 mm/ < 6 mm. For each category: number of cases/number of cases with progression. *p* value refers to the log-rank test for equality of survival distribution

Patients with iTFD < 6 mm had significantly reduced progression-free survival (univariable hazard ratio (HR) of 2.36, *p* < 0.001; Table 3 and Fig. 5) and so had patients with DOI ≥ 5 mm (HR = 2.19, *p* = 0.005), APD ≥ 17 mm (HR = 2.77, *p* < 0.001) and iDMI (HR = 2.95, *p* < 0.001). In multivariable models including patient age and preoperative high-risk histology (endometrioid grade 3/non-endometrioid histology based on curettage/biopsy), all the dichotomized MRI variables still had significant prognostic impact (Table 3). In the univariable models APD ≥ 17 mm and iDMI yielded highest concordance (concordance = 0.63 for both) with lower figures for iTFD < 6 mm (concordance = 0.60) and DOI ≥ 5 mm (concordance = 0.58), whereas in the multivariable models all imaging variables had similar concordance (concordance = 0.72–0.73) (Table 3).

### Interobserver agreement

**Fig. 6:**
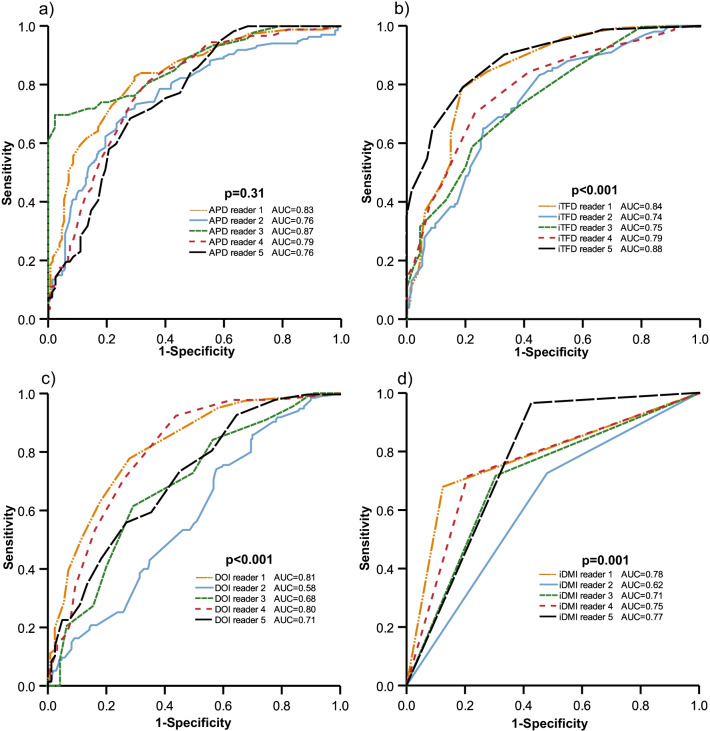
Receiver operating characteristic (ROC) curves for the different readers’ MRI tumor measurements for the prediction of pDMI (surgicopathologically deep myometrial invasion): **a** APD (anteroposterior diameter), (**b**) iTFD (tumor-free distance to serosa based on imaging findings), (**c**) DOI (depth of myometrial invasion) and (**d**) iDMI (presence of deep (≥ 50%) myometrial invasion based on standard imaging reading). *p* values refer to the test of equal AUC values across tumor measurements

The interobserver reproducibility for the MRI measurements was excellent for APD and very good for iTFD yielding ICCs of 0.87 and 0.73, respectively, whereas for DOI interobserver reproducibility was poor with ICC of 0.37 (Table 4). For the dichotomized imaging variables, the overall interobserver agreement was good for APD ≥ 17 mm with *κ* = 0.80, moderate for iTFD < 6 mm (*κ* = 0.59) and only fair for iDMI (*κ* = 0.41) and DOI ≥ 5 mm (*κ* = 0.29) (Table 4). For the prediction of pDMI, APD yielded similar area under the ROC curves (AUC) (*p* = 0.31), whereas iTFD, DOI and iDMI yielded significantly different AUCs for the five readers (*p* ≤ 0.001 for all; Fig. 6).

## Discussion

In this large MRI study of EC patients, we found that the preoperative tumor marker iTFD yielded best diagnostic performance for the prediction of pDMI at surgicopathological staging. Furthermore, the cutoff (≥ / < 6 mm)-derived iTFD marker outperformed other imaging markers for predicting pDMI. Thus, iTFD represents a very promising imaging marker for improved preoperative prediction of pDMI and high-risk disease in endometrial cancer.

Short pTFD based on hysterectomy specimen has previously been proposed as a reliable and accurate marker for the prediction of lymph node metastases, high-risk histological subtype and poor outcome in EC [16–19]. Non-uniform international guidelines for histological reporting of hysterectomy specimen in EC have led to variable and changing reporting standards for the different tumor measurements when assessing pDMI [20–23]. At present, no guidelines include pTFD as an obligate tumor measurement, although listed as optional by three guidelines [20, 22, 23]. In the present cohort, pTFD based on hysterectomy specimen was not routinely reported.

pDMI based on hysterectomy specimen is routinely assessed, defines FIGO stage 1B and is considered one of the strongest predictors of hematogenous spread and aggressive disease in EC [24]. Standardization in the assessment of pDMI by measuring pTFD, DOI and percentage of MI has been attempted, however, with variable results [16–18, 25–27]. We found that the interobserver variability for MRI-assessed iTFD was lower than for iDMI and DOI, suggesting that iTFD is a more reproducible imaging marker. The interobserver agreement for iDMI was only fair (overall *κ* = 0.41) in the present study, with *κ*-value being within the range of that reported previously (0.32–0.84) [9, 28, 29]. Interestingly, for the dichotomized tumor measurements iTFD (≥ / < 6 mm) and APD (< / ≥ 17 mm) the agreement was moderate (*κ* = 0.59) and very good (*κ* = 0.80), respectively, whereas it was only fair (*κ* = 0.29) for DOI (< / ≥ 5 mm). No previous studies have reported numbers for interobserver agreement for iTFD or DOI measurements based on preoperative imaging. The very good agreement for APD is supported by a study reporting excellent agreement for imaging-based tumor size measurements in EC [10].

The limitations in interobserver reproducibility between radiologists for preoperative imaging markers are to some extent shared by pathologists for the assessment of corresponding markers based on hysterectomy specimen. Discordant findings for pDMI have been reported in 20–33% of EC cases [25, 30]. In two small studies (*n* = 177 and *n* = 50, respectively), the agreement was very good and good for diagnosing pDMI (κ of 0.82–84 and 0.75, respectively) [31, 32]. In one of these studies (comparing seven pathologists) they discovered better agreement for pTFD (≤ 1.75 mm/(≤ 7 mm)/[≤ 10 mm]; *κ* = 0.77/(0.73)/[0.69]) than for DOI (≥ 4 mm; *κ* = 0.59) [32]; which is in line with our findings that iTFD is more reproducible than DOI based on MRI. The pathologists also rated pTFD to be easier to measure than pDMI and DOI [32]. Importantly, the radiologists individually select the image slice for tumor measurements, whereas the pathologic assessment is based on predefined (one to two) slides [32], which will inherently favor better agreement among pathologists.

In the present study, we found that iTFD < 6 mm was the optimal cutoff for predicting pDMI, yielding a sensitivity of 76% and a specificity of 77%. No previous reports have explored the value of iTFD measurements based on preoperative MRI in EC. However, one study using 3D ultrasound measurements in EC proposed a cutoff of iTFD ≤ 9 mm for predicting pDMI (*n* = 96) [33], which yielded higher sensitivity (100%) than that based on our proposed cutoff (iTFD < 6 mm), however, at the cost of lower specificity (61%).

In the present study, APD ≥ 17 mm was the only independent predictor of poor survival. The prognostic impact of tumor size is supported by previous studies linking increasing tumor size to poor outcome [10, 34, 35]. This finding is likely to be due to the well-known increased virulence and metastagenicity characterizing most large malignant tumors originating at various sites [34].

This study has some limitations. It is a single-center study and the MRI examinations were performed at two different scanners. The 3 T protocol was, however, intentionally set up to be very similar to the 1.5 T protocol and it seems unlikely that the use of different magnetic field strengths has substantially biased our results. The MRI readings were performed by radiologists with varying experience with pelvic MRI, ranging from two years to more than ten years’ experience. Although this poses a possible limitation, such an approach is more likely to reflect the standard diagnostic setting in which MR images are being read in daily routine. Information recorded in the routine pathology reports were used in this study without a pathologic review by several pathologists. Since limitations in interobserver reproducibility for pathologic assessment are also likely to exist, this should ideally have been included; however, this was not feasible in the present study. In future research, comparison of MRI-assessed iTFD with measuring pTFD in the hysterectomy specimen with multiple observers could be of potential interest.

### Conclusion

The preoperative MRI-based measurement iTFD to serosa yielded highest area under the ROC curve for the prediction of deep myometrial invasion, and iTFD < 6 mm is associated with poor prognosis in endometrial cancer. Thus, iTFD to serosa at MRI may represent a valuable adjunct to routinely reported imaging markers for preoperative prediction of deep myometrial invasion and high-risk disease in endometrial cancer.

### Clinical relevance/application

Preoperative measurement of tumor-free distance to serosa at MRI with cutoff value < 6 mm represents a promising marker for identifying deep myometrial invasion and high-risk disease in endometrial cancer.

## Supplementary Information

**Additional file 1: Supplementary table 1:** MRI scanning protocols used in the project.  **Supplementary table 2:** Imaging measurements and imaging parameters recorded by the three radiologists based on preoperative MRI. **Supplementary table 3:** Pairwise comparison of sensitivity, specificity and accuracy with McNemars test. **Supplementary table 4:** Sensitivity, specificity and accuracy for the prediction of pDMI by the preoperative MRI markers iTFD < 6 mm, DOI ≥ 5 mm, APD ≥ 17 mm and iDMI based on 1.5T (*n* = 279) and 3T (*n* = 71) MRI.

## Data Availability

In accordance with European and Norwegian regulations, we are prohibited from releasing the original patient imaging data, as the individual consent form did not explicitly allow open sharing of patient sensitive data (applies to both imaging and clinical patient data).

## Abbreviations

APD: Anteroposterior tumor diameter
DOI: Depth of myometrial tumor invasion
EC: Endometrial cancer
iDMI: Deep myometrial invasion (DMI) based on imaging findings
iTFD: Tumor-free distance to serosa (TFD) based on imaging findings
pDMI: Deep myometrial invasion (DMI) based on pathology findings
pTFD: Tumor-free distance to serosa (TFD) based on pathology findings
