# Supplementary material for: MRI-assessed tumor-free distance to serosa predicts deep myometrial invasion and poor outcome in endometrial cancer
Source: Insights Imaging. 2022 Jan 8;13:1. doi: 10.1186/s13244-021-01133-z (PMC8742796; doi:10.1186/s13244-021-01133-z)
Supplement: Supplementary file 1 — Additional file 1: Supplementary table 1: MRI scanning protocols used in the project. Supplementary table 2: Imaging measurements and imaging parameters recorded by the three radiologists based on preoperative MRI. Supplementary table 3: Pairwise comparison of sensitivity, specificity and accuracy with McNemars test. Supplementary table 4: Sensitivity, specificity and accuracy for the prediction of pDMI by the preoperative MRI markers iTFD < 6 mm, DOI ≥ 5 mm, APD ≥ 17 mm and iDMI based on 1.5T (n = 279) and 3T (n = 71) MRI. [file 13244_2021_1133_MOESM1_ESM.docx]

**ELECTRONIC SUPPLEMENTARY MATERIAL**

| **Supplementary table 1: MRI scanning protocols used in the project** | | | | | | | | |
| --- | --- | --- | --- | --- | --- | --- | --- | --- |
| MR scanner | Sequence | Plane | TR/TE_1_/TE_2_ (ms) | FA  (deg) | Slice thickness/ Intersection gap (mm) | Acquisition Matrix | FOV(mm) | Pixel size (mm) |
| 1.5T  Siemens Avanto | T2 TSE  T2 TSE  DWI  FS T1VIBE  FS T1 VIBE +C | AxObl  SAG  AxObl  AxObl  AxObl | 6310/95  4920/95  3100/79  7.23/2.55  7.23/2.55 | 150  150  90  20  20 | 3.0/0.3  3.0/0.3  5.0/1.0  2.0/0  2.0/0 | 256x205  256x205  128x128  192x154  192x154 | 180x180  180x180  300x300  250x250  250x250 | 0.9x0.7  0.9x0.7  2.3x2.3  1.6x1.3  1.6x1.3 |
| 3T  Siemens Skyra | T2 TSE  T2 TSE  DWI RESOLVE  T1 VIBE DIXON  T1 VIBE DIXON +C | AxObl  SAG  AxObl  AxObl  AxObl | 4330/94  7360/101  6010/74/126  5.86/2.46/3.69  5.86/2.46/3.69 | 150  160  180  9  9 | 3.0/0.3  3.0/0.3  3.0/0.3  1.2/0  1.2/0 | 326x384  310x320  144x144  139x256  139x256 | 200x200  200x200  200x200  250x250  250x250 | 0.5x0.5  0.6x0.6  1.4x1.4  1.0x1.0  1.0x1.0 |
| C= intravenous contrast (2min post contrast), deg=degrees, DWI=diffusion weighted imaging, FA=flip angle, FLASH=Fast Low Angle SHot, FOV=field of view, FS=fat saturated, mm=millimeter, ms=millisecond, AxObl= axial oblique slice orientation, RESOLVE=REadout Segmentation Of Long Variable Echo trains, SAG=sagittal slice orientation, TE=echo time, TR=repetition time, TSE=turbo spin echo, VIBE=Volumetric Interpolated Breath-hold Examination | | | | | | | | |

| **Supplementary table 2: Imaging measurements and imaging parameters recorded by the three radiologists based on preoperative MRI** |
| --- |
| Anteroposterior tumor diameter (APD), mm |
| Depth of myometrial invasion (DOI), mm |
| Tumor free distance to serosa (iTFD), mm |
| Deep (≥50%) myometrial invasion (iDMI) (yes/no) |

| **Supplementary table 3: Pairwise comparison of sensitivity, specificity and accuracy with McNemars test** | | | |
| --- | --- | --- | --- |
|  | Sensitivity | Specificity | Accuracy |
| iTFD <6mm vs DOI ≥5mm | 76% vs 86%, p=0.06 | 77% vs 49%, **p<0.001** | 77% vs 63%, **p<0.001** |
| iTFD <6mm vs APD ≥17mm | 76% vs 78%, p=0.73 | 77% vs 69%, **p=0.045** | 77% vs 73%, p=0.20 |
| iTFD <6mm vs iDMI | 76% vs 76%,  p=1.0 | 77% vs 73%, p=0.18 | 77% vs 74%, p=0.26 |
| DOI ≥5mm vs APD ≥17mm | 86% vs 78%, p=0.11 | 49% vs 69%, **p<0.001** | 63% vs 73%, **p=0.001** |
| DOI ≥5mm vs iDMI | 86% vs 76%, **p=0.02** | 49% vs 73%, **p<0.001** | 63% vs 74%, **p<0.001** |
| APD ≥17mm vs iDMI | 78% vs 76%, p=0.70 | 69% vs 73%, p=0.38 | 73% vs 74%, p=0.73 |
| iTFD, tumor free distance to serosa based on imaging findings; DOI, depth of invasion; APD, anteroposterior tumor diameter and  iDMI, deep (≥50%) myometrial invasion based on standard imaging reading  All significant p-values are given in boldface | | | |

| **Supplementary table 4: Sensitivity, specificity and accuracy for the prediction of pDMI by the preoperative MRI markers iTFD <6mm, DOI ≥5mm, APD ≥17 mm and iDMI based on 1.5T (n=279) and 3T (n=71) MRI** | | | |
| --- | --- | --- | --- |
| Sensitivity, % (no. of patients) | 1.5T | 3T | p-value ^α^ |
| Sensitivity,iTFD<6mm* | 73% (77/105) | 85% (29/34) | p=0.17 |
| Sensitivity, DOI≥5mm* | 81% (85/105) | 100% (34/34) | **p=0.004** |
| Sensitivity, APD≥17mm* | 78% (82/105) | 79% (27/34) | p=1 |
| Sensitivity, iDMI⁺ | 71% (75/105) | 91% (31/34) | **p=0.02** |
|  |  |  |  |
| Specificity, % (no. of patients) |  |  |  |
| Specificity, iTFD<6mm* | 77% (134/174) | 73% (27/37) | p=0.67 |
| Specificity, DOI≥5mm* | 50% (87/174) | 43% (16/37) | p=0.47 |
| Specificity, APD≥17mm* | 68% (118/174) | 76% (28/37) | p=0.43 |
| Specificity, iDMI⁺ | 72% (125/174) | 76% (28/37) | p=0.69 |
|  |  |  |  |
| Accuracy, % (no. of patients) |  |  |  |
| Accuracy, iTFD<6mm* | 76% (211/279) | 79% (56/71) | p=0.64 |
| Accuracy, DOI≥5mm* | 62% (172/279) | 70% (50/71) | p=0.21 |
| Accuracy, APD≥17mm* | 72% (200/279) | 77% (55/71) | p=0.37 |
| Accuracy, iDMI⁺ | 72% (200/279) | 83% (59/71) | p=0.05 |
|  |  |  |  |
| APD, anteroposterior tumor diameter; DOI, depth of invasion; iDMI, deep myometrial invasion (DMI) based on imaging findings; pDMI, DMI based on pathology findings; iTFD, tumor free distance to serosa based on imaging findings  *Optimal cut-off values for iTFD, DOI and APD based on the receiver-operating-characteristics analysis (Youden index) for prediction of pDMI in hysterectomy specimen  ⁺Presence of deep (≥50%) myometrial invasion based on standard imaging reading  ^α^Fisher’s exact test, p-value represents difference in sensitivity, specificity and accuracy between 1.5T and 3T for each MRI marker.  All significant p-values are given in boldface | | | |
